# The feasibility of progressive resistance training in women with polycystic ovary syndrome: a pilot randomized controlled trial

**DOI:** 10.1186/s13102-016-0039-8

**Published:** 2016-05-11

**Authors:** Lisa Vizza, Caroline A. Smith, Soji Swaraj, Kingsley Agho, Birinder S. Cheema

**Affiliations:** School of Science and Health, Western Sydney University, Locked Bag 1797, Penrith, NSW 2751 Australia; The National Institute of Complementary Medicine, Western Sydney University, Penrith, NSW 2751 Australia; Department of Endocrinology, Concord Repatriation General Hospital, Concord West, NSW 2138 Australia

**Keywords:** Weight training, Exercise, Quality of life, Menstrual cyclicity, Psychological health

## Abstract

**Background:**

To evaluate the feasibility of executing a randomized controlled trial of progressive resistance training (PRT) in women with polycystic ovary syndrome (PCOS).

**Methods:**

Women with PCOS were randomized to an experimental (PRT) group or a no-exercise (usual care) control group. The PRT group was prescribed two supervised and two unsupervised (home-based) training sessions per week for 12 weeks. Feasibility outcomes included recruitment and attrition, adherence, adverse events, and completion of assessments. Secondary outcomes, collected pre and post intervention, included a range of pertinent physiological, functional and psychological measures.

**Results:**

Fifteen participants were randomised into the PRT group (*n* = 8) or control group (*n* = 7); five women (*n* = 2 in PRT group and *n* = 3 in control group) withdrew from the study. The most successful recruitment sources were Facebook (40 %) and online advertisement (27 %), while least successful methods were referrals by clinicians, colleagues and flyers. In the PRT group, attendance to supervised sessions was higher (95 %; standard deviation ±6 %) compared to unsupervised sessions (51 %; standard deviation ±28 %). No adverse events were attributed to PRT. Change in menstrual cycle status was not significantly different between groups over time (*p* = 0.503). However, the PRT group significantly increased body weight (*p* = 0.01), BMI (*p* = 0.04), lean mass (*p* = 0.01), fat-free mass (*p* = 0.005) and lower body strength (*p* = 0.03), while reducing waist circumference (*p* = 0.03) and HbA_1c_ (*p* = 0.033) versus the control group. The PRT group also significantly improved across several domains of disease-specific and general health-related quality of life, depression, anxiety and exercise self-efficacy.

**Conclusion:**

A randomized controlled trial of PRT in PCOS would be feasible, and this mode of exercise may elicit a therapeutic effect on clinically important outcomes in this cohort. The success of a large-scale trial required to confirm these findings would be contingent on addressing the feasibility hurdles identified in this study with respect to recruitment, attrition, compliance, and collection of standardized clinical data.

**Trial registration:**

Australia New Zealand Clinical Trials Registry; ACTRN12614000517673 Registered 15 May 2014.

## Background

Polycystic ovary syndrome (PCOS) is a common endocrine disease that affects 9-18 % of women [1]. Hyperandrogenism, menstrual irregularity and polycystic ovaries define the condition [2], and common features include insulin resistance, hirsutism, acne, alopecia, and markers of cardiometabolic disease risk (e.g. android obesity, inflammation, dyslipidemia, etc.) [3, 4]. PCOS is a leading cause of oligo/anovulation and oligo/amenorrhea, infertility, and miscarriages [5, 6]. Depression [7], anxiety [8], body image difficulties [9] and low health-related quality of life (HRQoL) [10] are common in this population.

Clinical trials in PCOS have shown that aerobic training (e.g. cycling, walking) or high-intensity interval training prescribed for 12-24 weeks can significantly improve important clinical outcomes, including insulin sensitivity, body fat percentage, total and LDL-cholesterol, c-reactive protein (CRP), and psychological outcomes (e.g. HRQoL and depression) [11–17]. Such studies have informed clinical practice guidelines, which recommend that women with PCOS engage in ≥90 min of aerobic training weekly [18].

Progressive resistance training (PRT) is the most potent exercise modality for improving skeletal muscle mass and quality [19, 20]. Notably, studies have consistently shown that PRT can counteract metabolic diseases, including insulin resistance [20–23]. This is a key consideration for women with PCOS given that insulin resistance is implicated in the etiology of the disease [24]. The myogenic adaptations induced by PRT are often accompanied by a range of physiological (metabolic), functional and psychological adaptations that may be clinically important in this cohort [19]. PRT is currently recommended within exercise prescription guidelines for healthy adults and those with other major chronic diseases, including type 2 diabetes [25–31]. However, PRT is not currently recommended within clinical practice guidelines for PCOS [18].

Only two studies to date have investigated the isolated effect of PRT in women with PCOS [32–34]. These studies have shown that chronic PRT (10-16 weeks) can significantly improve several clinically important outcomes in this cohort, including body composition, circulating androgens, blood glucose, sexual dysfunction, depression and anxiety [32–34]. However, there is currently a need for additional research on the safety and efficacy of PRT in PCOS utilizing robust study designs. Therefore, the purpose of the present pilot study was to evaluate the feasibility of a executing a randomized controlled trial (RCT) of PRT in women with PCOS to inform the development of a large-scale clinical trial.

## Methods

### Study design

This study randomized participants into an experimental (PRT) group or a no-exercise (usual care) control group. Randomization assignments were generated via www.randomization.com by an investigator not involved in data collection, and given to participants in sealed envelopes upon the completion of baseline testing. The Human Research Ethics Committee at Western Sydney University (reference H10448) approved all procedures, and written informed consent was obtained from all participants.

### Participants and recruitment

Eligibility Criteria: (1) Age 18-42 years with a diagnosis of PCOS (confirmed via the participant’s general practitioner or specialist); (2) not currently participating in PRT; (3) not pregnant nor breastfeeding; (4) no history of cardiovascular, kidney, respiratory disease, uncontrolled hypertension or cancer; (5) no use of cigarettes for >6 months; (6) no acute or chronic medical condition that would make assessment and interventions potentially hazardous or any of the outcomes impossible to assess; (7) cognition and English language sufficient to understand research procedures and provide informed consent; and (8) willingness to be randomised and undergo study protocols. Participants were recruited by the lead investigator (L.V.) from February 20 to September 21, 2014 (~7.5 months.). Recruitment was done via flyer advertisements, social media advertisement (i.e. Facebook), free online advertisement (i.e. Gumtree), referrals from local clinicians, word of mouth, referrals by a research colleague who had conducted PCOS research, and a database from a previous PCOS study on the use of herbal medicine.

### Feasibility outcomes

#### Recruitment and attrition

Recruitment (or referral) source data was obtained by asking each potential participant upon initial contact how she first became aware of the study. One source was recorded per potential participant. Recruitment rate was computed by dividing the number of women randomized by the recruitment period (~7.5 months). Participant attrition was monitored throughout the study, and reasons for attrition were sought from all participants who withdrew.

#### Adherence

Adherence to the PRT intervention, including supervised and home-based components, was recorded using log-books and computed as the number of sessions attended divided by the number of sessions offered, multiplied by 100 %.

#### Adverse events

Adverse events in both groups were recorded using a structured questionnaire administered weekly, in person in the PRT group and via telephone/email in the control group.

#### Completion of assessments

The clinical outcomes assessments completed at baseline (week 0) and follow-up (week 13), and weekly status checks to monitor adverse events, were recorded for each participant by the lead investigator (L.V) at the university campus and/or a private clinic.

### Clinical outcomes

Clinical outcomes were assessed prior to and following the 12-week intervention period (week 0 and 13). Week 13 testing was completed >72 h after the final exercise session in the PRT group.

#### Physiological outcomes

Participants retrospectively reported on the duration of their last menstrual period at baseline (week 0) and that of their most recent period at follow-up (week 13). During the study, menstrual cyclicity was self-monitored by participants using a standardized menstrual diary. Menstrual cycle was categorized as amenorrhea (no period for >199 days) [35], oligomenorrhea (cycle duration of 35 to 199 days) [35] or normal cycle (cycle duration of 23 to 35 days) [36]. Improvement or worsening of menstrual cyclicity was defined as a change in the menstrual cycle category from week 0 to week 13.

Height and weight were measured using calibrated equipment and standard procedures [37]; body mass index (BMI) was derived from these measures. Waist circumference was measured at the level of the umbilicus [38]. Body composition was analyzed using dual-energy X-ray absorptiometry (DEXA) as previously described [39]; measurements were obtained for fat mass, lean (muscle) mass, fat-free mass and body fat percentage.

Blood samples were obtained and analyzed through a private pathology lab (Douglas Hanly Moir) the morning following an 8-h overnight fast. Biochemical assays were obtained for: glycosylated haemoglobin (HbA_1c_), insulin, glucose, high-sensitivity C-reactive protein (CRP), testosterone, and sex-hormone binding globulin (SHBG) using standard assays (coefficient of variation (CV): 0.5 % to 6 %). Free androgen index was calculated by [testosterone (mmol/litre) / SHBG (mmol/litre) x 100], and HOMA-2 was calculated by an online computer model available at www.OCDEM.ox.ac.uk.

#### Functional outcomes

Upper and lower body maximum isometric strength was assessed using triceps extension and knee extension positions, respectively, using a portable electronic dynamometer (Chatillon DFX-II, AMETEK, Paoli, PA; CV 9.4 %) as previously described [40].

#### Psychological outcomes

Disease-specific HRQoL was evaluated using the *Polycystic Ovary Syndrome Questionnaire* (PCOSQ), a 26-item scale which assesses 5 domains: emotions, body hair, weight, infertility problems and menstrual problems [41]. Domain scores can range from 1 to 7 with higher scores representing higher quality of life. The *Medical Outcomes Trust Short Form-36* (SF-36) was used to evaluate eight domains of general HRQoL (i.e. Physical Functioning, Role Physical, Bodily Pain, General Health, Vitality, Social Functioning, Role Emotional and Mental Health) [42]. Higher scores, ranging from 0-100, denote higher HRQoL. The *Depression, Anxiety and Stress Scale 21* (DASS-21) was used to evaluate symptoms of (1) depression (i.e. hopelessness, low self-esteem, and low positive affect) (2) anxiety (i.e. autonomic arousal, musculoskeletal symptoms, situational anxiety and subjective experience of anxious arousal) and (3) stress (i.e. tension, agitation, and negative affect). Higher scores in each scale denote more severe symptoms. The *Exercise Self-Efficacy Scale* was used to measures the self-efficacy of individuals to undertake physical exercise [43]. Scores can range from 10 to 40 with higher scores indicating higher self-efficacy.

#### Intervention

Participants in the PRT group were prescribed two supervised training sessions per week on non-consecutive days (i.e. Monday, Wednesday or Friday) for 12 weeks at the university campus. The PRT group also performed two home-based (unsupervised) exercise sessions consisting of lower-intensity calisthenics to facilitate habitual movement and behaviour change [44]. Supervised sessions lasted for approximately 60 min, and included a standardized (5 min) warm-up and cool-down on exercise cycle or treadmill. PRT exercises included lat pulldown, leg curl, seated row, leg press, calf raise, chest press, split squat, shoulder press, biceps curl, triceps extension and abdominal curl. All sets (except abdominal curl) were performed to neuromuscular fatigue, i.e. 8-12 repetitions maximum; loads were increased with strength gains. Two sets of each exercise were prescribed in the first 2 weeks of training. From week 3, all exercises except split squats and shoulder press were progressed to 3 sets.

The home-based calisthenics exercises were undertaken on non-PRT days and included lying external hip rotations (‘clam shells’), side leg raises, push-ups on knees, wall squats, oblique curls, core stabilization exercises (‘bird dog’ and abdominal hollowing), performed for 3 sets x 10 repetitions each. Participants received a different set of callisthenic home-based exercises every four weeks. Participants were asked to record the number of repetitions of each exercise performed in a log book. This record was collected weekly.

### Control

Participants in the control group did not receive any PRT intervention and were instructed to continue with their current lifestyle and usual healthcare and medical treatments.

### Statistical analyses

All available data were included, regardless of patient compliance to the intervention in an intention-to-treat analysis, performed using the Statistical Package for the Social Sciences (IBM© SPSS©, Version 22.0). Data from patients unavailable for follow-up assessments at week 12 were carried forward from baseline. Baseline characteristics were compared using t-tests and chi-square, as appropriate. Within group changes over time were evaluated by paired t-tests. Group x time effects were determined by analysis of covariance of the post-treatment score controlling for the baseline score for all continuous variables, and by chi square for change (improvement or worsening) of menstrual cyclicity. Furthermore, sensitivity analyses were completed without imputation of missing data, and using parametric and bootstrap method (10,000 replicates of the original sample size) to estimate the effect sizes, and the 95 % confidence interval (CI). A p value of <0.05 was considered as indicative of statistical significance.

## Results

### Feasibility outcomes

#### Recruitment and attrition

**Fig. 1:**
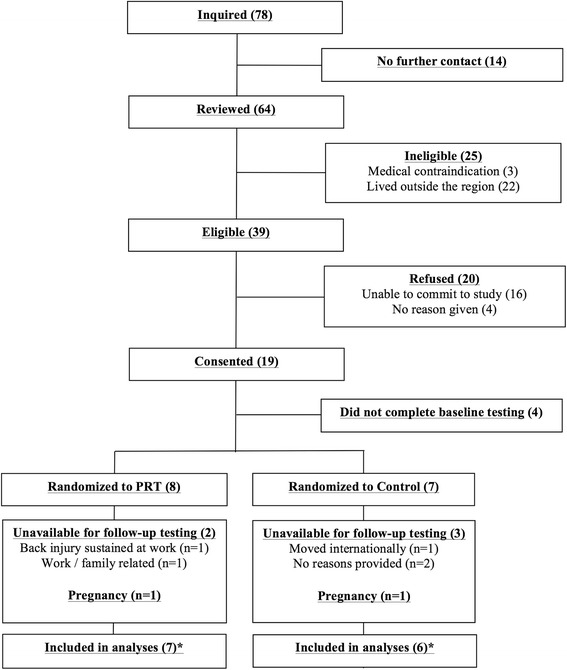
Participant flow chart. *Baseline data carried forward for two participants in the PRT and three participants in the control group that did not complete follow-up testing. Data for one pregnancy in each group excluded from the analyses

Seventy-eight women contacted the principal investigator regarding participation and received information about the study (Fig. 1). Fourteen women were not reviewed due to their lack of further contact. Sixty-four women were reviewed for eligibility. Of these women, 25 were deemed ineligible primarily due to living outside the local region (*n* = 22) (20 of these women were referred by Facebook). Thirty-nine women met the eligibility criteria for the study and 20 of them refused participation with the main reason provided being ‘lack of time’ (*n* = 16). Nineteen (*n* = 19) women consented and four (*n* = 4) did not complete baseline testing due to other commitments (*n* = 1) or refusal to be randomized to a no-exercise control group (*n* = 3). Fifteen women (*n* = 15) completed baseline testing and were randomized into the PRT group (*n* = 8) or the control group (*n* = 7) resulting in a randomization-to-screening percentage of 23 % (15 randomized / 64 reviewed). Of these women, the main sources of recruitment were Facebook (*n* = 6, 40 %) and online advertisement (via Gumtree) (*n* = 4, 26.6 %), followed by referrals by local clinicians (*n* = 3, 20 %), a research colleague (*n* = 1, 6.7 %) and flyers (*n* = 1, 6.7 %). Two women in the PRT group and three women in the control group withdrew from the study and were unavailable for follow-up testing, with reasons presented in Fig. 1. Two women (one per group) became pregnant during the trial and were excluded from analyses due to the confounding effect of pregnancy on the clinical outcomes. Thirteen women were included in the statistical analyses. Recruitment rate averaged two women per month (15 participants / 7.5 months).

#### Adherence to PRT

Adherence to training inclusive of the two participants in the PRT group who withdrew from training (Fig. 1) was 76 % ± 13 % for supervised training, 43 % ± 26 % for home-based (unsupervised) calisthenics training, and 60 % ± 10 % overall. Excluding these two participants, attendance was 95 % ± 6 % for supervised training, 51 % ± 28 % for unsupervised training and 73 % ± 6 % overall.

#### Adverse events

One participant in the PRT group sustained an ankle sprain (week 10) from a fall outside of training. This participant continued to train with two adjustments of the program to accommodate the injury (i.e. leg press exercise was performed unilaterally using the non-affected leg and knee extensions were substituted for the split squat). Another participant in the PRT group sustained a lower back injury at work in week 4, which resulted in withdrawal and inability to complete follow-up testing (Fig. 1). No adverse events were attributed to PRT. No adverse events were reported by any participant in the control group.

#### Completion of assessments

All participants in the PRT group (*n* = 5) and control group (*n* = 3) available for follow-up testing completed all clinical outcomes assessments (Fig. 1). All weekly status checks (100 %) were completed in person by the PRT group (*n* = 5), while the control group (via telephone/email) completed only 58 %.

### Baseline characteristics

There were no significant differences between groups at baseline, according to the descriptive characteristics presented in Table 1. However, trends were noted for waist circumference (*p* = 0.06) and hip circumference (*p* = 0.10). The age and BMI of participants ranged from 18-40 years and 18.3 kg/m^2^ to 54.6 kg/m^2^, respectively. In the total cohort, six participants had oligomenorrhea, four had amenorrhea, and three had a regular cycle at baseline. The most common prescription medication was metformin (*n* = 4 in the PRT group and *n* = 1 in the control group) and one participant in each group was prescribed an oral contraceptive.

**Table 1:** Baseline characteristics of the total cohort and groups

Characteristic	Total Cohort (*n* = 13)	PRT Group (*n* = 7)	Control Group (*n* = 6)
Age (y)	27 (5)	26 (7)	29 (3)
Height (cm)	163.8 (9.3)	168.2 (4.6)	158.7 (11.1)
Body weight (kg)	102.9 (35.0)	117.4 (36.6)	86 (27.0)
Body mass index (kg/m^2^)	37.8 (11.4)	41.3 (12.5)	34.0 (9.4)
Waist circumference (cm)	110.8 (27.2)	123.6 (29.0)	96.0 (16.3)
Hip circumference (cm)	124.1 (22.1)	133.5 (22.7)	113.0 (17.0)
Waist-to-hip ratio	0.88 (0.09)	0.92 (0.08)	0.85 (0.10)
Systolic blood pressure (mmHg)	116 (9)	114 (8)	118 (10)
Diastolic blood pressure (mmHg)	76 (6)	76 (7)	76 (6)
Menstrual cycle status (n):			
Normal cycle	3	2	1
Oligomenorrhea	6	2	4
Amenorrhea	4	3	1

Data reported as mean (standard deviation) except menstrual cycle status (n)

### Clinical outcomes

#### Physiological outcomes

Menstrual cyclicity improved from baseline in three women (one in the PRT group and two in the control group). Menstrual cyclicity worsened in one woman in the PRT group and remained unchanged in the other nine women in the study. Change in menstrual cycle status was not significantly different between groups (*p* = 0.503).

The PRT group reported a significant increase in body weight (*p* = 0.01) and BMI (*p* = 0.04) compared to the control group (Table 2). There was also a significant reduction in waist circumference (*p* = 0.03) and a significant increase in lean mass (*p* = 0.01) and fat-free mass (*p* = 0.005), indicating that the weight gain was due to muscle hypertrophy. There were no differences in fat mass or percent body fat between groups over time.

**Table 2:** Summary of within and between group changes on clinical outcomes

Outcome Measure	PRT (*n* = 7)			Control (*n* = 6)			*P* (between groups)	Effect Size (95 % CI)^a^
	Week 0	Week 12	*P*	Week 0	Week 12	*P*		
Physiological outcomes								
Body weight (kg)	117.4 (36.6)	118.9 (35.4)	0.06	86.0 (26.7)	86.0 (26.8)	0.77	0.01	0.49 [0.61,3.83]
Body mass index (kg/m^2^)	41.3 (12.5)	41.7 (12.1)	0.12	33.8 (9.4)	33.8 (9.4)	N/A	0.04	0.37[0.04,1.20]
Waist Circumference (cm)	123.6 (29.0)	121.5 (29.1)	0.04	95.9 (16.3)	96.6 (17.2)	0.20	0.03	0.40^b^
Fat mass (kg)	58.6 (23.3)	59.0 (22.7)	0.50	39.6 (18.3)	40.6 (19.2)	0.24	0.59	0.03[-2.96,1.79]
Lean mass (kg)	54.7 (12.5)	56.0 (11.3)	0.16	43.9 (10.4)	43.0 (10.0)	0.23	0.01	0.49^b^
Fat-free mass (kg)	57.4 (13.6)	58.8 (11.9)	0.13	46.8 (10.8)	46.0 (10.4)	0.26	0.005	0.57^b^
Percent body fat (%)	49.7 (8.4)	49.6 (7.6)	0.84	45.2 (10.7)	46.1 (11.2)	0.22	0.36	0.08[-2.66,1.06]
HbA1c (%)	5.3 (0.4)	5.1 (0.3)	0.037	5.1 (0.3)	5.2 (0.4)	0.24	0.03	0.39^b^
Fasting insulin (mU/L)	21 (11)	20 (12)	0.49	9 (5)	10 (6)	0.18	0.24	0.14[-5.50,1.55]
Fasting glucose (mmol/L)	4.7 (0.8)	4.9 (0.7)	0.03	4.8 (0.4)	4.9 (0.4)	0.71	0.18	0.17[-0.08,0.40]
HOMA-2	2.62 (1.33)	2.56 (1.43)	0.72	1.19 (0.64)	1.24 (0.71)	0.19	0.27	0.11[-0.65,0.20]
hsCRP (mg/L)	8.9 (10.7)	8.0 (10.8)	0.38	3.6 (5.1)	3.9 (5.4)	0.12	0.36	0.09[-3.57,1.41]
Testosterone (nmol/L)	1.5 (0.3)	1.7 (0.5)	0.38	1.7 (0.4)	1.8 (0.3)	0.11	0.91	0.00[-0.44,0.49]
SHBG (nmol/L)	32 (26)	27 (22)	0.39	47 (25)	47 (25)	0.36	0.25	0.13[-22.58,6.50]
Free androgen index (%)	8.6 (7.3)	9.8 (6.6)	0.26	4.5 (2.7)	4.7 (2.7)	0.11	0.18	0.17[-0.82,3.83]
Functional outcomes								
Upper body strength	190.2 (42.5)	211.5 (52.1)	0.10	141.3 (53.0)	134 (51.0)	0.12	0.06	0.33^b^
Lower body strength	258.7 (67.2)	313.2 (90.3)	0.04	221.8 (59.5)	214.8 (64.9)	0.08	0.03	0.45^b^
Psychological outcomes								
PCOSQ								
Emotions	4.4 (0.6)	5.1 (0.5)	0.02	3.2 (1.0)	3.1 (0.9)	0.13	0.003	0.60[0.53,1.95]
Body hair	4.9 (2.0)	4.5 (1.5)	0.37	4.2 (1.7)	4.2 (1.9)	1.0	0.58	0.03[-1.22,0.71]
Weight	3.3(1.7)	4.1(1.7)	0.06	2.4 (1.5)	2.3 (1.5)	0.70	0.04	0.35[0.03,1.796]
Infertility problems	0.9 (0.4)	3.5 (0.9)	0.01	1.1 (0.6)	1.9 (1.2)	0.11	0.03	0.41^b^
Menstrual problems	4.1 (0.8)	3.9 (0.9)	0.52	3.0 (1.4)	3.0 (1.4)	N/A	0.92	0.00[-1.06,0.96]
SF-36								
Physical Functioning	83.0 (10.4)	89.3 (12.4)	0.05	94.2 (5.8)	90.8 (10.7)	0.18	0.02	0.42^b^
Role Physical	21.4 (4.9)	22.3 (3.3)	0.36	17.7 (7.3)	18.8 (7.9)	0.70	0.70	0.02[-4.58,6.58]
Bodily Pain	67.3 (20.0)	65.3 (18.0)	0.55	45.2 (28.3)	48.8 (22.0)	0.36	0.87	0.00[-10.46,9.00]
General Health	49.0 (22.3)	54.3 (25.2)	0.27	53.3 (14.4)	49.2 (10.7)	0.19	0.14	0.20[-3.73,22.58]
Vitality	47.3 (26.2)	56.2 (21.3)	0.12	35.4 (26.4)	30.2 (22.9)	0.29	0.02	0.45^b^
Social Functioning	75.0 (14.4)	89.3 (15.2)	0.08	60.4 (18.4)	52.1 (9.4)	0.24	0.002	0.64^b^
Role Emotional	18.0 (5.8)	23.0 (4.1)	0.10	9.7 (8.2)	8.3 (7.5)	0.36	0.009	0.51^b^
Mental Health	71.4 (22.6)	84.2 (17.2)	0.06	51.7 (20.7)	46.7 (20.7)	0.31	0.009	0.52^b^
DASS-21								
Depression	10.8 (6.8)	5.4 (5.4)	0.050	12.7 (9.6)	14.7 (9.0)	0.20	0.01	0.50^b^
Anxiety	10.3 (5.6)	7.4 (7.0)	0.082	12.3 (11.2)	14.3 (10.8)	0.11	0.03	0.41^b^
Stress	12.6 (7.9)	9.7 (9.3)	0.220	21.7 (10.9)	23.0 (11.2)	0.33	0.17	0.18 [-11.11,2.25]
Exercise Self Efficacy Scale	29.4 (4.7)	31.3 (4.2)	0.095	32.5 (5.3)	30.0 (4.0)	0.10	0.04	0.37^b^

^a^ = parametric between groups effect sizes and their confidence intervals

^b^ = sample size too small to estimate the confidence intervals

Data reported as mean (standard deviation). Abbreviations: *HbA1c* hemoglobin A1c, *HOMA-2* Homeostatic model of assessment 2, *CRP* c-reactive protein, *SHBG* sex hormone binding globulin, *PCOSQ* Polycystic Ovary Syndrome Questionnaire, *SF-36* Medical Outcomes Trust Short Form-36, *DASS-21* Depression, Anxiety and Stress Scale 21, Effect size = Cohen’s d

The PRT group reported a significant reduction in HbA_1c_ over time compared to the control group (*p* = 0.03). Unexpectedly, within group analysis revealed that the PRT group reported a significant increase in fasting glucose (*p* = 0.03). No other physiological adaptations to PRT were noted.

#### Functional outcomes

The PRT group experienced a trend toward increased upper body strength (*p* = 0.06) and a significant increase in lower body strength (*p* = 0.03) compared to the control group.

#### Psychological outcomes

The PRT group significantly improved three of five PCOSQ domain scores compared to the control group: emotions (*p* = 0.003), weight (*p* = 0.04) and infertility problems (*p* = 0.03) versus the control group. In addition, the PRT group reported a significantly improvement in five of eight HRQoL (SF-36) domain scores (physical functioning [*p* = 0.02], vitality [*p* = 0.02], social functioning [*p* = 0.002], role emotional [*p* = 0.009] and mental health [*p* = 0.009)], two DASS-21 domains (depression [*p* = 0.01] and anxiety [*p* = 0.03]), and exercise self-efficacy (*p* = 0.04) compared to the control group. No other psychological group x time effects were noted.

### Post hoc analyses

To determine the effect of higher adherence on the PRT group, *post hoc* analyses were performed using data from participants who completed >75 % of the supervised sessions (and >60 % of the total training sessions, i.e. supervised plus home-based) (*n* = 5). The outcomes of these analyses did not differ from the primary analyses.

### Sensitivity analyses

Group x time analyses were performed without imputation of missing data from participants that did not undergo final assessment (i.e. per protocol). The outcomes of these analyses did not differ with the primary analyses, except that group x time interaction effects for the following outcomes became non-significant: body weight (*p* = 0.07), BMI (*p* = 0.15), and lean mass (*p* = 0.07). Using both parametric and bootstrap method, our result indicated that the confidence intervals of effect sizes cross each other, indicating that the two methods did not differ statistically and hence, the effect sizes and their CI’s for only the parametric method was reported.

## Discussion

This pilot study evaluated the feasibility of a PRT intervention in women with PCOS within an RCT to inform the development of a robust clinical trial. Our findings suggest that it is feasible to investigate PRT in women with PCOS, and that this anabolic intervention may induce a number of clinically important adaptations.

The study enrolled approximately two participants per month over 7.5 months. This rate of recruitment is similar to studies prescribing aerobic training in women with PCOS (0.5 to 3 women per month) [45–49]. However, the duration of recruitment in these studies was longer (8-40 months) resulting in larger sample sizes (*n* = 20 to *n* = 124) [45–49]. Other studies have not stated the duration of recruitment [14–16, 50–52]. Clearly, participant recruitment is a challenging aspect of clinical research [53], and strategies for enhancing recruitment are therefore important. Our most successful recruitment methods were the social media platform Facebook (*n* = 6, 40 %) and online advertisement via Gumtree (*n* = 4, 26.6 %). The least successful strategies included referral by local clinicians (*n* = 3, 20 %), research colleagues (*n* = 1, 6.7 %) and flyers (*n* = 1, 6.7 %). Having the support of local clinicians is essential to fostering participant recruitment in clinical trials [54]. However, clinicians are often busy with their own professional duties and may not be able to offer the level of support required [55]. To foster recruitment via clinicians, it may be necessary to engage in regular meetings and/or create more streamlined pathways for referral.

Although Facebook was our most effective recruitment method, it also resulted in a large number of ineligible women contacting the principal investigator. Future studies conducted within small geographic regions should consider more targeted methods of advertising via Facebook (i.e. paid advertisement to more effectively reach the local community). Alternatively, studies conducted across multiple geographical sites or countries may benefit from using Facebook for wider-scale recruitment. No previous study of exercise in PCOS has noted the use of Facebook as a recruitment method [14, 16, 45, 48–51].

Our participant attrition rate was 38 % overall (i.e. 29 % (2/7) in the PRT group and 50 % (3/6) in the control group). Previous studies of exercise training in PCOS have reported participant attrition rates ranging from 25 % to 45 % [14, 45, 47–49, 51], while other studies have not provided these data [46, 48, 52]. In the present study, two women in the control group were lost immediately after randomization, while three other women consented and withdrew from the study given their concerns about randomization. It has been suggested that participants who are randomized to a no-treatment control group, or a treatment group contrary to their desire, may not accept randomization and refuse participation [56]. Strategies to minimize participant attrition may include the use of a placebo-control (i.e. unloaded or non-progressive training) or a waitlist control group. Moreover, future studies could opt to investigate the additive effect of PRT to an exercise intervention prescribed according to current guidelines which emphasize aerobic training [17, 18], i.e. by comparing a group receiving PRT plus aerobic training to a group receiving aerobic training only.

Attendance to supervised training sessions in the present study was high (95 ± 6 %). High attendance rates to supervised training (>80 %) have been noted in many trials of aerobic training in PCOS [16, 46, 52]. By comparison, attendance to the home-based sessions in the present study was lower (51 %). A previous PCOS study which prescribed unsupervised exercise (i.e. walking) also demonstrated a lower compliance rate (43 %) [48]. Studies in other cohorts have reported both high [57] and low compliance to home-based training [58]. Potential reasons for low compliance to home-based exercise prescriptions may include lack of motivation or time [59]. Future studies should consider possible strategies to foster adherence to unsupervised training, including SMS reminders, effective time management strategies (e.g. diarizing sessions into a routine schedule), motivational cues and rewards [60–62], and other behavior strategies [63–65].

All reported adverse events were not related to the study intervention indicating that PRT is safe intervention for women with PCOS. This finding is consistent with the evidence base in other chronically diseased cohorts, including type 2 diabetes [66–70].

In general, the completion of clinical outcomes assessments was satisfactory in the present study. However, the administration of weekly status checks via email and telephone to monitor adverse events in the control group proved problematic with only 58 % completed. This may be improved by arranging weekly visits with participants, if feasible.

The PRT intervention applied in the present study yielded a number of clinically important adaptations that may be associated with better disease management and treatment of the underlying pathology. No significant change in menstrual cyclicity was noted in the PRT or control group. However, studies of aerobic training [47, 52] and dietary and exercise intervention, including those involving aerobic plus PRT [51] have shown improved menstrual cyclicity using similar methodology (i.e. self-reported menstrual diary). There is reason to hypothesize that PRT can improve menstrual cyclicity in women with PCOS [24]. Therefore, future studies should continue to investigate this endpoint in women with oligo/amenorrhea, including the dosages of PRT required to induce adaptation. Such investigations require vigilant monitoring of menstrual cycles (i.e. date of last menstrual period in women with amenorrhea and documenting menstrual cycle length in women with oligomenorrhea and regular cycles) and reporting of confounding variables including physical activity and dietary habits.

PRT may counteract the etiology of PCOS through its effect on body composition [24]. Improvements in skeletal muscle size and quality secondary to PRT in type 2 diabetes have been accompanied by reductions in visceral fat [67, 71] and improvements in insulin sensitivity and glucoregulation [67, 71–73]. Increased insulin sensitivity and glucoregulation, in turn, may downregulate androgen synthesis and hyperandrogenemia in women with PCOS, which could counteract the disease process (i.e. premature growth arrest of follicles) and menstrual irregularity [24]. The anthropometric changes experienced by the PRT group in the present study, including the reduction in waist circumference (*p* = 0.03) and increase in fat-free mass (*p* = 0.005) were accompanied by an improvement in HbA1c (*p* = 0.031) versus the control group, indicating parallel improvement of body composition and glucoregulation. However, the PRT group experienced no change in HOMA-2, several key endocrine markers (i.e. free androgen index [*p* = 0.18], testosterone [*p* = 0.91], SHBG [*p* = 0.246]) or other key haemotological markers associated with PCOS (i.e. CRP, fasting insulin) versus the control group (Table 2). These physiological data are difficult to interpret within a small-scale study. Future studies are required to investigate these adaptations, including the determination of dose-response effects for each outcome. These studies would also benefit from using more sensitive measures of insulin resistance, including the euglycaemic-hyperinsulaemic clamp [74], and gold-standard assessment of steroids involving liquid chromatography-tandem mass spectrometry [75] with the standardisation of assessments to a particular phase of the menstrual cycle (i.e. day 2-5 of menstrual cycle), if required.

Unexpectedly, within group analyses showed that the PRT group increased fasting glucose over time (*p* = 0.03). This finding may be reflective of an acute effect, i.e. in response to psychological stress or lack of sleep. Previous studies in PCOS, type 2 diabetes and/or obesity that prescribed aerobic, mixed training, and/or diet plus exercise have reported significant reductions in fasting insulin and fasting glucose [46, 47, 52, 76–79] and therefore this outcome requires further exploration in PCOS.

The PRT group demonstrated a trend toward increased upper body strength (*p* = 0.06) and a significant rise in lower body strength (*p* = 0.03) compared to the control group. PRT is the modality of choice for eliciting strength gains [80, 81] and these adaptations are typically accompanied by improvements in performance-based tests and psychological attributes, including HRQoL [40, 82, 83]. Women with PCOS have been shown to have greater muscular strength than their healthy peers [84] and future studies are required to determine the clinical importance of strength adaptation in this cohort.

The PRT group improved three of five PCOSQ domains versus the control group: emotions (*p* = 0.003), weight (*p* = 0.04) and infertility problems (*p* = 0.03). In addition, the PRT group significantly improved five of eight HRQoL (SF-36) domain scores (physical functioning [*p* = 0.02], vitality [*p* = 0.02], social functioning [*p* = 0.002], role emotional [*p* = 0.009] and mental health [*p* = 0.009]), DASS-21 domains for depression (*p* = 0.01) and anxiety (*p* = 0.03), and exercise self-efficacy (*p* = 0.035) versus the control group. The psychological benefits of exercise or physical activity in women with PCOS have been noted previously and are clinically relevant [85, 86]. Future studies involving placebo-control groups are required to determine if these psychological adaptations can be attributed solely to the PRT, or are influenced by the social interaction with trainers and/or other participants.

Strengths of this study include the collection of feasibility outcomes, and the inclusion of important clinical outcomes. Limitations of the study included the small sample size (*n* = 15) and lack of monitoring and controlling for key confounding variables such as physical activity and diet.

## Conclusion

In summary, a randomized clinical trial of PRT in PCOS would be feasible to conduct, and this mode of exercise may elicit a therapeutic effect on a range of pertinent outcomes in this cohort. A suitably powered clinical trial is required to confirm these findings and answer novel research questions in relation to prescribing PRT as a therapeutic intervention in PCOS. The success of a large-scale trial required to confirm these findings would be contingent on addressing the feasibility hurdles identified in this study with respect to recruitment, attrition, compliance, and collection of standardized clinical data.

## Ethics approval and consent to participate

The Human Research Ethics Committee at Western Sydney University (reference H10448) approved all procedures, and written informed consent was obtained from all participants.

## Availability of data and materials

The data and materials can be made available on request via contacting the corresponding author.

## Abbreviations

BMI: body mass index
CI: confidence interval
CRP: C-reactive protein
DASS-21: depression, anxiety and stress scale 21
DEXA: dual-energy X-ray absorptiometry
HbA1c: glycosylated haemoglobin
HRQoL: health-related quality of life
PCOS: polycystic ovary syndrome
PCOSQ: polycystic ovary syndrome questionnaire
PRT: progressive resistance training
RCT: randomized controlled trial
SF-36: medical outcomes trust short form-36
SHBG: sex-hormone binding globulin
